# Archaeosome Adjuvant Overcomes Tolerance to Tumor-Associated Melanoma Antigens Inducing Protective CD8^+^ T Cell Responses

**DOI:** 10.1155/2010/578432

**Published:** 2011-01-18

**Authors:** Lakshmi Krishnan, Lise Deschatelets, Felicity C. Stark, Komal Gurnani, G. Dennis Sprott

**Affiliations:** National Research Council of Canada, Institute for Biological Sciences, Ottawa, ON, Canada K1A 0R6

## Abstract

Vesicles comprised of the ether glycerolipids of the archaeon *Methanobrevibacter smithii* (archaeosomes) are potent adjuvants for evoking CD8^+^ T cell responses. We therefore explored the ability of archaeosomes to overcome immunologic tolerance to self-antigens. Priming and boosting of mice with archaeosome-antigen evoked comparable CD8^+^ T cell response and tumor protection to an alternate boosting strategy utilizing live bacterial vectors for antigen delivery. Vaccination with melanoma antigenic peptides TRP_181-189_ and Gp100_25-33_ delivered in archaeosomes resulted in IFN-**γ** producing antigen-specific CD8^+^ T cells with strong cytolytic capability and protection against subcutaneous B16 melanoma. Targeting responses against multiple antigens afforded prolonged median survival against melanoma challenge. Entrapment of multiple peptides within the same vesicle or admixed formulations were both effective at evoking CD8^+^ T cells against each antigen. Melanoma-antigen archaeosome formulations also afforded therapeutic protection against established B16 tumors when combined with depletion of T-regulatory cells. Overall, we demonstrate that archaeosome adjuvants constitute an effective choice for formulating cancer vaccines.

## 1. Introduction

Constant immunosurveillance against tumors both in experimental mouse models and cancer patients suggests that immunotherapy can be an effective way of controlling many forms of cancer. For example, absence of the cytokine IFN-gamma or CD8^+^ T cells and perforin leads to aggressive or even spontaneous tumor development [1–3]. The identification of many T cell-defined tumor antigens over the past two decades has lead to the logical search for a reliable “widely applicable” cancer vaccine. Indeed the demonstration in both mice and humans that T cells of defined antitumor specificity can be generated and can eliminate cancers has been very encouraging [4, 5]. Nevertheless, strategies have been limiting in their ability to sufficiently break tolerance and induce high avidity T cells against cancer self-antigens. Cancer vaccination as opposed to vaccination against infection poses a unique challenge in that the immune activation has to occur in the absence of “danger signal” recognition. Such a response is often weak, does not sustain for long periods, and leads to tolerance and regulatory T cell induction [6]. 

Vaccine adjuvants offer one potential solution to circumventing the weak response to cancer antigens. The molecular definition of danger-associated molecular patterns (DAMPs) has provided a rational choice of immune modulators such as CpG and other TLR-ligand agonists [7, 8]. These have been explored as add-ons in vaccine formulations for boosting cancer vaccine efficacy. Another approach includes heterologous prime-boost containing different types of vaccine adjuvants that are often able to increase the breadth and potency of the immune response [9, 10]. However, the short half-life of small molecule immunomodulators and potential toxicity of DAMPs often limit their widespread use. Delivery systems including liposomes, virus-like particles, microspheres, and ISCOMATRIX adjuvants provide alternative options for formulating diverse antigenic and adjuvant components and facilitating specific immunity with minimal damaging side-effects [11, 12]. 

The polar membrane lipids of *Archaea* are characteristic of this *Domain* of life in having isoprenoid chains of constant length, with novel stereochemistry of ether bond linkages to *sn-*2, 3 carbons of the glycerol backbone [13]. We have developed a potent vaccine adjuvant delivery system constituted by these polar lipids, termed “Archaeosomes” [11]. Antigen may be entrapped within the hydrophilic core of the vesicles or anchored in the membrane or linked to surface-exposed groups akin to antigen-loading principles for liposomes. Our lead Archaeosome type is composed of the total natural mixture of polar lipids extracted from the methanogen, *Methanobrevibacter smithii *(Ms archaeosomes). The superior features of Archaeosomes relative to conventional liposomes and other adjuvants are recruitment and activation of dendritic cells *in vivo*, ability to direct antigen cargo for MHC class I processing leading to potent induction of CD8^+^ T cell response, and stability of archaeal lipid cores facilitating profound immune memory [14]. Most importantly, these archaeosomes bypass TLR-2- and IL-12-mediated signaling for activating CD8^+^ T cells [15]. 

Thus, we have evaluated the ability of archaeosomes to effectively break self-tolerance to native melanoma antigens and afford tumor protection. We show that archaeosomes can provide strong antitumor immunity to self-antigens and that a prime-boost strategy with alternate adjuvants was without benefit.

## 2. Materials and Methods

### 2.1. Materials

Ovalbumin grade VI, 2-mercaptoethanol, RBC lysing solution, carboxyfluorescein, AEC chromogen kit, and PKH26 red fluorescent cell linker kit were from Sigma-Aldrich (Sigma-Aldrich Canada Ltd., Oakville, Ontario, Canada). Flow cytometry antibodies were purchased from BD Biosciences, RPMI 1640 medium and gentamicin from Invitrogen Life Technologies, FBS from HyClone, G418 from Calbiochem, and murine recombinant IL-2 from ID Labs. Peptides H-2K^b^ restricted Ova_257–264 _(SIINFEKL) and HLA.A2/H-2K^b^ TRP-2_180–188 _(SVYDFFVWL), CTL epitope from tyrosinase-related protein-2 [16], Gp100_25–33_ (KVPRNQDWL) from human melanoma antigen Gp100 [17] were synthesized in-house. Live recombinant *Mycobacterium bovis *(BCG-OVA) and *Listeria monocytogenes* (LM-OVA) expressing OVA_257–264 _were constructed as described previously [18].

### 2.2. Preparation of Antigen-Loaded Archaeosomes

Peptide-loaded archaeosome formulations were prepared from the total polar lipids (TPLs) extract from *Methanobrevibacter smithii *(Ms) [19]. GP100_25–33_ or TRP-2_181–189_ was entrapped separately, or a coentrapped preparation was prepared where both peptides were loaded simultaneously. For model antigen studies the whole protein Ovalbumin (OVA) was entrapped in Ms archaeosomes. 

A dry lipid film containing 20 mg TPL was hydrated at 35°C in 0.5 mL of filter-sterilized Milli-Q water containing 5 mg of OVA. In the case of peptides, the hydration was done in a high pH environment, and the proportion of the peptide was diminished to avoid aggregate formation between the charged peptide and the negatively charged polar lipids. 100 mM Triethanolamine (TEA) pH 9 containing 2 mg peptide was added to the dry lipids. Assessment of archaeosome integrity was done microscopically using phase contrast at 2000X magnification. Archaeosomes were bath sonicated for 1–3 min to reduce the vesicles diameter to 100–200 nm and were annealed overnight at 4°C for membrane stabilization. 

Nonentrapped antigen was removed by ultracentrifugation at 207,000 × g (*r*_average_) for 2 h (Beckman centrifuge). The liposome pellet was washed three times with 8 mL of pyrogen-free filter-sterilized Milli-Q water. The final pellet was resuspended in 1.0 ml of water and filtered through 0.45 *μ*m filters.

### 2.3. Archaeosome Characterization

Gaussian, number-weighted size distributions were monitored with a Nicomp particle sizer Model 370, Santa Barbara, CA. All archaeosome vaccines used herein ranged between 80 and 118 nm average diameter. Entrapment efficiency was determined based on the dry weight of a known aliquot and quantification of the incorporated antigen. Ovalbumin content was evaluated through SDS-PAGE gel electrophoresis and densitometry of bands revealed by Coomassie blue staining. Peptide amounts were assayed by RP-HPLC using a Zorbax C-18 reverse-phase column (150 × 4.6 mm) with a guard cartridge installed in a DX-300 Dionex dual piston HPLC system (Sunnyvale, CA). The peptides were eluted at a flow rate of 1 mL/min using a gradient aqueous mobile phase from 2% acetonitrile in 0.1% TFA to 70% acetonitrile in 0.085% TFA over 60 min and revealed by UV absorbance at a 216 nm wavelength. Integration was done by a Dionex 4290 integrator. Quantification was done using a calibration curve based on known amounts of each of the respective peptides.

### 2.4. Mice and Immunizations

Inbred, 6–8-week-old female C57BL/6J mice were obtained from the Jackson Laboratory (Bar Harbor, ME) and maintained in the Animal facility of the Institute for Biological Sciences, NRC, in accordance with guidelines from the Canadian Council on Animal Care. Mice were injected subcutaneously (0.1 mL volume) at the base of the tail, with antigen-archaeosomes (Ms-antigen), antigen in PBS (no adjuvant), or live recombinant preparations LM-OVA or BCG-OVA as per doses indicated in figure legends. All final archaeosome preparations were in PBS prior to the immunizations. Antigen-archaeosome immunization scheme was based on peptide amounts of 1, 10, 15, 20, or 30 *μ*g in 0.1 mL. Each archaeosome formulation was named according to the antigen entrapped as Ms-OVA, Ms-Gp100, and Ms-TRP. Archaeosome preparations with coentrapped Gp100 and TRP peptides (both peptides within the same archaeosomes) were named Ms-Gp100-TRP co-entrap, whereas the ones named Admix were defined by the combination prior to injection of two (Ms-Gp100 + Ms-TRP admix) of the singly entrapped peptides. For comparison purposes, the admixed archaeosome immunization scenarios were based on the same antigen amount as directed by the coentrapped version. Refer to figure legends for loading shown as *μ*g antigen/mg dry weight of the different immunization cocktails.

### 2.5. Cell Lines

EL-4 (H-2^b^) was obtained from the American Type Culture Collection (ATCC, Rockville, MD) and maintained in RPMI 1640 medium (Life Technologies, Grand Island, NY) supplemented with 2-mercaptoethanol, 8% FBS (HyClone, Logan, UT) and 10 *μ*g/ml gentamicin (Life Technologies). B16 melanoma cells were cultured in RPMI plus 8% FBS. B16OVA cells, expressing the gene for OVA, were obtained from Dr. Edith Lord (University of Rochester, NY) and cultured in RPMI plus 8% FBS, additionally containing 400 *μ*g/mL G418.

### 2.6. Assessment of Numbers of Antigen-Specific CD8^+^ T Cells In Vivo

The activation of antigen-specific CD8^+^ T cells after immunization with Ms-OVA, LM-OVA, and BCG-OVA was tracked *in vivo* using the tetramer assay. Briefly, peripheral blood lymphocytes were incubated in 200 *μ*L PBS plus 1% BSA (PBS-BSA) with anti-CD16/32 at 4°C. After 10 min., cells were stained with PE-tetramer and anti-CD8 for 30 min. at room temperature. Cells were washed with PBS, fixed in 0.5% formaldehyde, and acquired on BD Biosciences FACS Canto analyzer. The tetramers used include H-2K^b^OVA_257–264_, H-2D^b^TRP-2_180–188_, and H-2K^b^Gp100_25–33_, all purchased from Beckman Coulter.

### 2.7. CTL Assays

The antigen-specific cytolytic activity of spleen cell effectors after recall stimulation with antigen was carried out as described in detail previously [20]. Briefly, spleen cells were cultured with 0.01 *μ*g/mL of the appropriate antigen (TRP2_181–189_ or Gp100_25–33_) for 5 days *in vitro*, and the ensuing effectors were used in a standard ^51^Cr-release CTL assay against nonspecific and antigen-specific targets. EL-4 cells served as the nonspecific target, whereas they were preincubated with 10 *μ*g/ml of the CTL peptide for 1 h to generate specific targets. The percent specific lysis at various effector:target ratios was calculated using the formula: [(cpm experimental-cpm spontaneous)/(cpm total-cpm spontaneous)] × 100. 

*In vivo* cytolytic activity of antigen-specific CD8^+^ T cells was enumerated according to the protocol of Barber et al. [21]. Donor spleen-cell suspensions from syngeneic mice were prepared and red blood cells lysed using trisbuffered ammonium chloride (RBC lysing solution). Cells were stained with the dye PKH26 (4 *μ*M) and split into two aliquots. One aliquot was stained with low concentration of CFSE (0.5 *μ*M) and incubated in R8 medium. The second aliquot was stained with 10X CFSE (5 *μ*M) and incubated with the appropriate CTL peptide (10 *μ*g/mL) in R8 medium. After 30 min. of incubation, the two aliquots were mixed 1 : 1 and injected (20 × 10^6^/mouse) into previously immunized recipient mice. PBS-injected recipient mice served as controls. At 24 h after the donor cell transfer, spleens were removed from recipients, single cell suspensions prepared, and cells analyzed by flow cytometry. The *in vivo* lysis percentage of peptide pulsed targets was enumerated according to previously published equation [21].

### 2.8. Enumeration of IFN-*γ* Secreting Cells

Enumeration of IFN-*γ* secreting cells was done by ELISPOT assay. Briefly, ELISPOT plates were coated with anti-IFN-*γ* antibody, blocked, and incubated with spleen cells in various numbers (in a final cell density of 5 × 10^5^/well using feeder cells) in the presence of IL-2 (0.1–1 ng/mL) and R8 media or the appropriate CTL epitope peptide (5–25 *μ*g/mL) for 48 h at 37°C, 8% CO_2_. The plates were then incubated with the biotinylated secondary antibody (37°C, 2 h) followed by avidin-peroxidase conjugate (room temperature for 1 h). Spots were revealed using 3-amino-9-ethylcarbazole (AEC chromogen kit).

### 2.9. Tumor Model

Mice were injected with 10^6^ B16-OVA or B16 tumor cells (in PBS plus 0.5% normal mouse serum) in the shaved lower dorsal region. From day 5 onwards, palpable solid tumors were measured using digital calipers. Tumor size, expressed in mm^2^, was obtained by multiplication of diametrically perpendicular measurements. Mice were euthanized when the tumor sizes reached a maximum of 300 mm^2^.

### 2.10. Statistical Analysis

Unpaired, Student's *t*-test was used to determine the statistical difference between two groups of data, whereas one-way ANOVA was used to compare multiple sets of data. Tumor survival curves were analyzed by log-rank test. The statistical package of the Graph Prism software was used for statistical analyses of all data.

## 3. Results

### 3.1. Priming and Boosting with Archaeosomes Confers Superior Quantity of Antigen-Specific CD8^+^ T Cell Response

**Figure 1:**
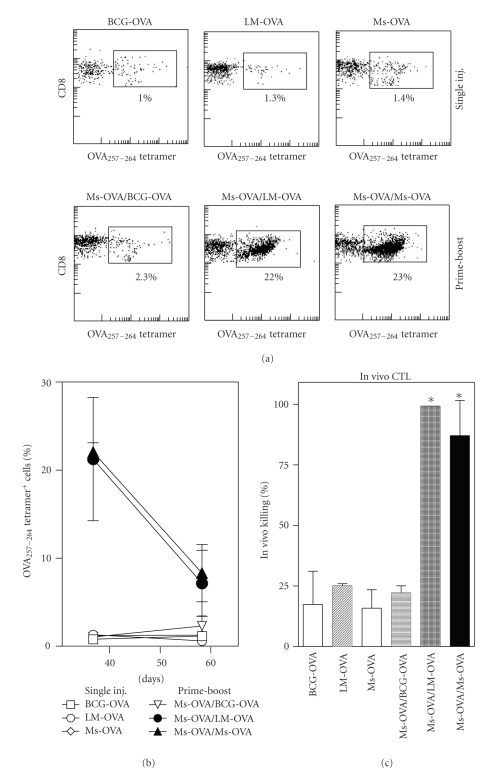
CD8^+^ T cell response after heterologous prime-boost with archaeosomes and live vectors. Mice were vaccinated (subcutaneously) with Ms-OVA (20 *μ*g OVA entrapped in Ms archaeosomes, 94.1 nm average size, 38 *μ*g/mg loading), or 10^4^ CFU of live LM-OVA or BCG-OVA. Prime-boost regimens involved the same Ms-OVA vaccine given on days 0 and 30, or heterologous boost on day 30 with the BCG-OVA or LM-OVA vector. The CD8^+^ T cell response was evaluated based on the percentage of OVA_257–264_ tetramer positive cells in the blood (a, b) and *in vivo* CTL response (c). (a) Representative scatter plots showing tetramer positive cells on day 7 after a single injection (top panel) or after prime-boost on day 37 (bottom panel). The square gate indicates percentage of tetramer positive cells in the blood of immunized mice. (b, c), Mean ± SD of 5 mice per group. *Response was statistically significant from single dose group by student's *t*-test (*P* < .05).

We have previously shown that Ms archaeosomes prime CD8^+^ T cell response to entrapped antigen. However, in many vaccination regimens, particularly for breaking tolerance, priming and boosting with alternate adjuvants, has been suggested to increase the magnitude and longevity of antigen-specific T cell response. We therefore evaluated whether boosting with a live vector may improve response to a primary Ms-OVA injection. Mice that received a single injection of BCG-OVA or LM-OVA evoked OVA-specific CD8^+^ T cells (based on OVA-tetramer binding) similar in numbers to those injected with particulate Ms-OVA on day 7 after immunization (Figure 1(a)). When Ms-OVA vaccination was followed by LM-OVA injection 30 days later, a clear increase in the number of antigen-specific CD8^+^ T cells was seen on day 37. However, the magnitude of the response was similar to priming and boosting with Ms-OVA (Figures 1(a) and 1(b)). In contrast, a BCG-OVA booster provided very little enhancement of the antigen-specific response primed by Ms-OVA. The *in vivo* CTL response to vaccination showed a similar trend (Figure 1(c)), with single dose vaccines yielding low level of specific killing, whereas boosting with Ms-OVA or LM-OVA yielded strong enhancement of CD8^+^ T cell cytolytic ability. Therefore, there was no benefit to using an alternate delivery vector for boosting.

### 3.2. Prime-Boost Vaccination Affords Long-Term Protection against Melanoma Challenge

**Figure 2:**
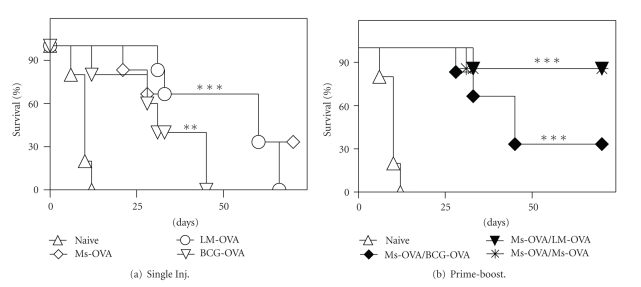
Tumor protection following heterologous prime-boost vaccination. C57BL/6J mice were vaccinated subcutaneously with a single dose of Ms-OVA (20 *μ*g OVA, 38 *μ*g/mg lipid loading, 94.1 nm average archaeosome size), LM-OVA (10^4^ CFU), or BCG-OVA (10^4^ CFU) or with a prime-boost regimen as indicated. Mice were challenged with subcutaneous B16-OVA tumors 4 weeks post vaccination. Survival plots are based on euthanizing animals upon reaching a maximum tumor size of 300 mm^2^ (*n* = 5/group). Survival curves for vaccinated groups were significantly different from naïve group by log-rank test (**P < .01, ***P < .001).

We next correlated the CD8^+^ T cell response evoked by various vaccination regimens to protection against subcutaneous melanoma challenge. Mice vaccinated with a single dose of Ms-OVA, LM-OVA, or BCG-OVA exhibited a median survival of 60, 60, and 31 days, respectively, following subcutaneous melanoma challenge (Figure 2(a)). In contrast, prime-boost regimens that involved priming with Ms-OVA followed by boosting with Ms-OVA or LM-OVA afforded superior protection, with >90% of vaccinated mice being tumor-free for indefinite periods. Boosting with BCG-OVA resulted in a median survival of 45 days, which was greater than a single injection regimen (Figure 2(b)). Thus, prime-boost vaccination afforded superior tumor protective responses, but alternating vector delivery systems had no further benefit.

### 3.3. Archaeosome Vaccines Break Tolerance to Self-Antigen Cargo

**Figure 3:**
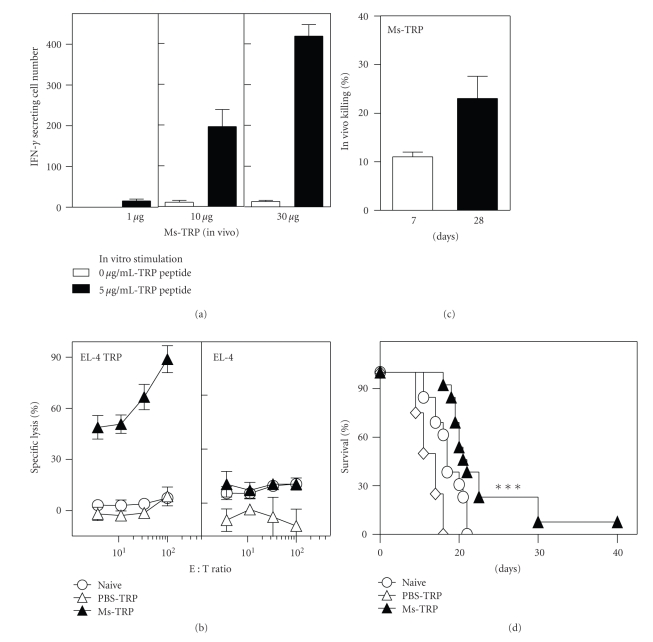
CD8^+^ T cell response and tumor protection induced by Ms-TRP vaccine. C57BL/6J mice were immunized in (a) with 1, 10, or 30 *μ*g TRP peptide entrapped in Ms archaeosomes on days 0 and 21. At 5 weeks, representative mice (*n* = 2 per group) were euthanized, the spleen cells were stimulated with IL-2 (0.1 ng/mL) and peptide (5 *μ*g/mL) for 48 h, and the frequency of IFN-gamma secreting cells was enumerated by ELISPOT. Mean ± SD (*n* = 3) of IFN-gamma secreting cells per 10^6^ spleen cells is indicated. Spleen cells from mice immunized with 10 *μ*g TRP-peptide per injection were also cultured for 5 days with antigen to generate CTL effectors. The ability of effectors to kill peptide specific (EL-4-TRP) versus nonspecific (EL-4) targets was evaluated in an in-vitro CTL assay (b). Mean killing ± SD of 2 mice per group at different effector : target ratio is indicated for Naïve, PBS-TRP, and Ms-TRP vaccinated mice (b). In another group of representative mice vaccinated with 20 *μ*g Ms-TRP, *in vivo* CTL response was evaluated on day 7 and day 28. Mean ± SD of *n* = 4 mice per group is indicated (c). Finally, groups of naïve (*n* = 12), Ms-TRP vaccinated (*n* = 12), and TRP-PBS (*n* = 4) vaccinated mice were challenged with B16 tumors at 6 weeks. Survival was monitored based on a maximum tumor size of 300 mm^2^. Tumor survival data are presented as an aggregate from 3 different experiments conducted, and TRP dose was 15–30 *μ*g/injection. Loading was 19 *μ*g peptide/mg lipid, and average archaeosome size was 99 nm. Survival with Ms-TRP is significantly different (*P* < .001) by log-rank test relative to naïve group.

**Figure 4:**
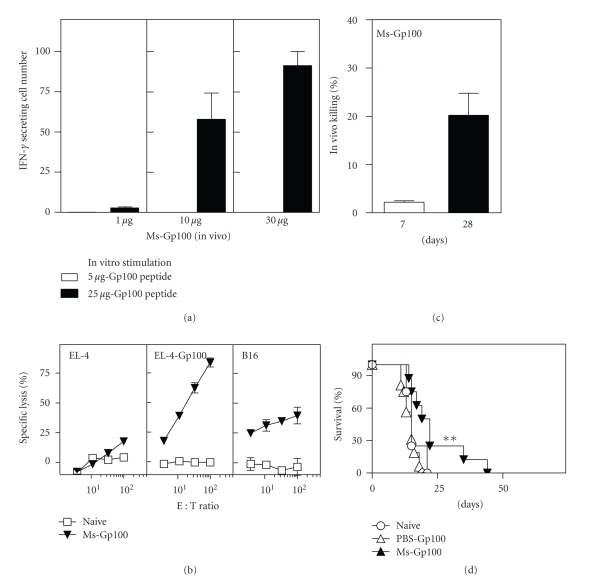
CD8^+^ T cell response and tumor protection induced by Ms-Gp100 vaccine. Mice were immunized with 1, 10, or 30 *μ*g Gp100 peptide entrapped in Ms archaeosomes at days 0 and 21. At 5 weeks, representative mice (*n* = 2 per group) were euthanized, the spleen cells were stimulated with IL-2 (0.1 ng/mL) and peptide (25 *μ*g/ml) for 48 h and the frequency of IFN-gamma secreting cells was enumerated by ELISPOT. Mean ± SD (*n* = 3) of IFN-gamma secreting cells per 10^6^ spleen cells is indicated. (a) At 5 weeks, *in vitro* CTL assay was also carried out on spleen cells of representative mice (*n* = 2 per group) immunized with 10 *μ*g Ms-Gp100 (b). Mean Killing ± SD of 2 mice per group at different effector : target ratio is indicated (b). In another group of representative mice vaccinated with 20 *μ*g Ms-Gp100, *in vivo* CTL response was evaluated on day 7 and day 28. Mean ± SD of *n* = 4 mice per group is indicated (c). Finally, response to subcutaneous B16 tumor challenge was evaluated at 6 weeks in groups of naïve (*n* = 12), Ms-Gp100 vaccinated (*n* = 12), and PBS-Gp100 (*n* = 4) vaccinated mice. Survival was monitored based on a maximum tumor size of 300 mm^2^. Tumor survival data are presented as an aggregate from 3 different experiments conducted, and Gp100 peptide vaccination dose ranged from 25 to 30 *μ*g/injection. Loading was 112 *μ*g peptide/mg archaeosomes and average size 94.3 nm. Survival for Ms-Gp100 group is significantly different (*P* < .05) from naïve mice by log-rank test.

As priming and boosting with model antigens entrapped in archaeosomes yielded a strong CD8^+^ T cell response and tumor protection, we next evaluated the response to self-antigen cargo delivered in archaeosomes. Immunization with 10 to 30 *μ*g of TRP-2_181–189_ peptide (H-2K^b^/HLA A.2 CD8^+^ T cell epitope) entrapped in archaeosomes evoked strong peptide-specific IFN-gamma production by CD8^+^ T cells as evaluated in an ELISPOT assay (Figure 3(a)). This correlated to a strong antigen-specific CTL response evoked in immunized mice both *in vitro* and *in vivo* (Figures 3(b) and 3(c)). Above all, immunized animals showed protection against a melanoma challenge (Figure 3(d)). Entrapment of Gp100_25–33_ (H-2K^b^/HLA A.2 CD8^+^ T cell epitope) peptide in archaeosomes also resulted in IFN-gamma production by CD8^+^ T cells (Figure 4(a)) and peptide-specific *in vitro* and *in vivo* CTL responses (Figures 4(b) and 4(c)). Additionally, the CD8^+^ T cells from vaccinated mice exhibited killing of B16 targets (not pulsed with peptide) *in vitro* in a standard chromium release killing assay (Figure 4((b)). It is likely that the weaker killing response against B16 targets relative to EL-4 peptide target may be attributable to lower endogenous expression of MHC-peptides complexes in B16 cells. Importantly, Gp100-archaeosome vaccination protected mice against subcutaneous melanoma challenge (Figure 4((d)). Although the responses to Gp100 were less dramatic than with TRP-2, archaeosomes were able in general to evoke functional CD8^+^ T cell responses to native melanoma antigens.

### 3.4. Archaeosome-Dual Antigen Formulations Evoke CTL Responses to Each Individual Antigenic Component

**Figure 5:**
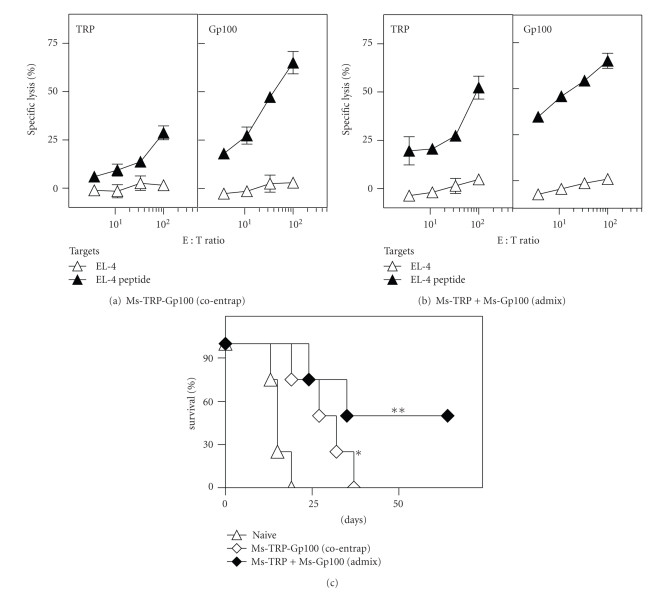
CD8^+^ T cell response and tumor protection induced by coentrapped melanoma peptide-archaeosome vaccine. Mice were vaccinated (days 0 and 21) with 25 *μ*g of peptides (coentrapped 5 *μ*g TRP and 20 *μ*g Gp100) or an equivalent admixed formulation of Ms-TRP and Ms-Gp100. *In vitro* CTL response of spleen effectors from representative mice (*n* = 2) was evaluated at 5 weeks (a, b) on TRP and Gp100 specific targets. Mean ± SD of triplicate cultures of effectors: targets at various ratios are indicated. At 6 weeks mice were challenged subcutaneously with B16 melanoma, and survival (*n* = 4/group) was evaluated based on a maximum tumor size of 300 mm^2^ (c). Loadings were 13 *μ*g Gp100 and 3 *μ*g TRP/mg archaeosomes for the coentrapped vaccine used in (a, c), and 60 *μ*g Gp100/mg lipid and 30 *μ*g TRP/mg archaeosomes for the admixed used in (b, c). Archaeosome size ranged from 104 to 110 nm. Survival for the vaccinated groups was significantly different compared to naïve animals by log-rank test (*P < .05; **P < .01).

While archaeosomes could evoke CTL responses to native melanoma antigens, the response to the individual antigenic component provided vaccinated animals with only a relatively short-lived protection to tumor challenge. We therefore coentrapped two melanoma CTL epitopes, TRP-2_181–189_ and Gp100_25–33_ within the same archaeosome vesicles. Following vaccination with a coentrapped antigen-archaeosome formulation, spleen cells of vaccinated mice exhibited CTL responses specific to both TRP-2 and Gp100 epitopes (Figure 5(a)). Nevertheless, the TRP-2-specific CTL response was weaker than that of Gp100, and this may be attributed to differential loading of the two respective peptides in the coentrapped formulation. A 25 *μ*g injection of peptides corresponded to 20 *μ*g Gp100 and 5 *μ*g TRP in 1.56 mg archaeosomes. However, vaccination with Ms-TRP and Ms-Gp100 archaeosomes admixed to achieve the equivalent 20 *μ*g Gp100 and 5 *μ*g TRP as in the coentrapped formulation, but this time entrapped in only 0.5 mg archaeosomes, resulted in a strong and comparable CTL activity being evoked against both antigens (Figure 5(b)). Consistent with the intensity of CTL responses, the coentrapped formulation afforded a median survival of only 29.5 days to tumor challenge, whereas immunization with the admixed Ms-TRP and Ms-Gp100 archaeosomes proved more effective at providing tumor protection (median survival of 49 days) (Figure 5(c)). Nevertheless, there was no statistical difference between the coentrapped and admixed vaccination group, and the median survival may be attributable to small sample size of the study. However, both groups demonstrated statistically significant difference from the control, nonvaccinated group. Therefore, admixed formulations simply provide a convenient means of controlling a more optimized dose and loading.

### 3.5. Targeting Dual Melanoma Antigens with an Admixed Archaeosome Formulation Affords Long-Term Tumor Protection

**Figure 6:**
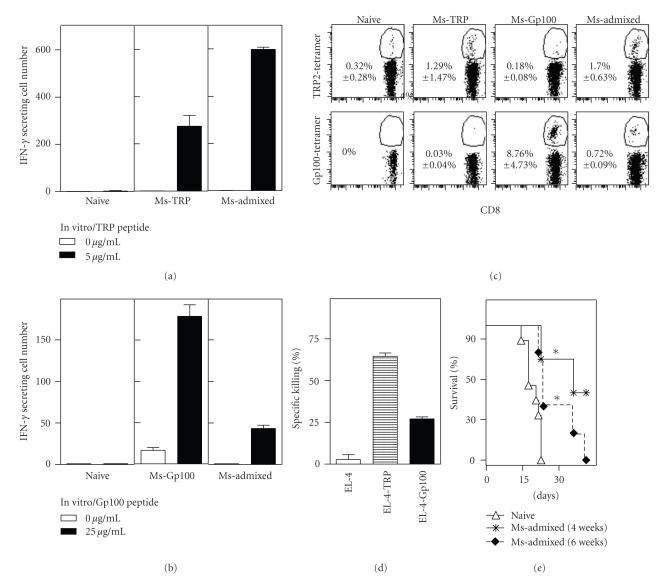
Optimized induction of CD8^+^ T cell response to dual melanoma antigen vaccine. Mice were vaccinated (days 0 and 21) with 30 *μ*g of individual peptide-archaeosome vaccines or in an admixed formulation containing 30 *μ*g of each peptide. At 4 weeks, representative mice (*n* = 3 per group) were euthanized, the spleen cells were stimulated with IL-2 (0.1 ng/mL) and each peptide (5–25 *μ*g/mL) for 48 h, and the frequency of IFN-gamma secreting cells was enumerated by ELISPOT. Mean ± SD (*n* = 3) of IFN-gamma secreting cells per 10^6^ spleen cells is indicated for TRP-peptide (a) and Gp100 peptide (b) stimulation. The percentage of tetramer-specific CD8^+^ T cells was enumerated in the blood on day 28. Representative plots for the various groups are shown (c), and the Mean ± SD (*n* = 3) of the response is indicated within each panel. *In vitro* CTL response in representative mice (*n* = 3/group) vaccinated with the admixed formulation was evaluated on day 28 against EL-4, EL-4-TRP, and EL-4-Gp100 targets (d). Mean ± SD at 100 : 1 effector : target ratio is indicated. At 4 or 6 weeks postvaccination, mice (*n* = 5 per group) were challenged with B16 melanoma (e). Survival curves for the vaccinated groups were significantly different from naïve by log-rank test (*P* < .05). Archaeosome loadings were 40 *μ*g Gp100/mg and 20 *μ*g TRP/mg lipid. Archaeosome size ranged from 110 to 117 nm.

In the above experiments, the admixed formulation of archaeosomes contained suboptimal amounts of melanoma antigens in order to serve as appropriate comparison to the coentrapped formulation. In order to test the full potential of targeting multiple melanoma antigens with archaeosome adjuvants, vaccination was carried out with a mixture of archaeosome melanoma peptide formulations such that each antigen was provided in an equivalent dose of 30 *μ*g, on day 0 and 21. Firstly, IFN-gamma production by CD8^+^ T cells, as evaluated in an ELISPOT assay on day 28 after vaccination, indicated a response against both TRP-2 and Gp100 peptide stimulations using the admixed formulation (Figure 6(a) and 6(b)). However, the antigen-specific response to Gp100 peptide was weaker when administered in an admixed formulation relative to Gp100-archaeosome by itself. Antigen-specific tetramer was also detectable in the blood on day 28 after priming against both peptides with the admixed formulation (Figure 6(c)). Here again, the endogenous response to Ms-Gp100 vaccine was stronger when administered by itself than in an admixed formulation. The CD8^+^ T cells evoked after admixed immunization effectively killed targets expressing TRP or Gp100 (Figure 6(d)), although the killing against TRP-expressing targets was stronger than the killing against Gp100 targets. Overall it appears that when coadministered, the TRP epitope may dominate in response over Gp100. Finally, we addressed the ability of the admixed vaccine to evoke tumor protection. In all previous experiments, tumor challenge was conducted at 6 weeks postimmunization, possibly during the declining phase of the T cell response. Therefore, we compared protection against tumors given at either 4 or 6 weeks postvaccination. Ms-Admixed formulation afforded significant protection (*P* < .01) when tumor challenge was carried out at either 4 or 6 weeks postvaccination (Figure 6(e)). Furthermore, there was no statistically significant difference in the protection seen at early (4 week) versus late (6 weeks) postvaccination (*P* = .4) suggesting ability of archaeosomes to afford memory responses to self-antigens. Comparing the aggregate data from several experiments, the median survival for TRP-2- or Gp100-archaeosomes single-peptide vaccine administered preventatively was 22 days relative to <17 days for naïve nonvaccinated mice. In contrast, targeting responses to both peptides using an admixed vaccine markedly improved median survival to 35 days.

### 3.6. Therapeutic Protection against Established B16 Tumors by Peptide-Archaeosome Vaccine

**Figure 7:**
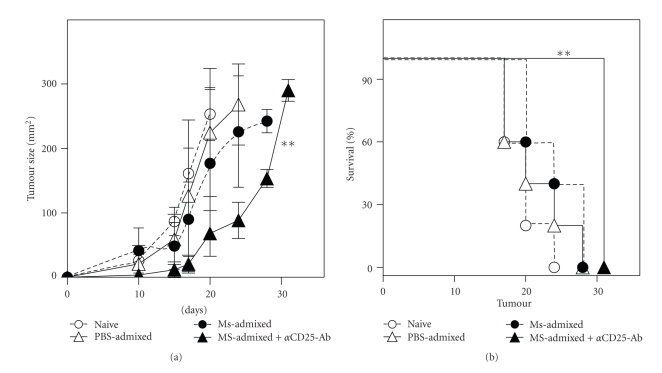
Therapeutic tumor protection by the dual melanoma antigen-archaeosome vaccine. Mice were injected with 10^5^ B16 melanoma cells subcutaneously on day 0. Vaccination with 30 *μ*g of each peptide in PBS (PBS-admixed) or as mixture of Ms-Gp100 and Ms-TRP (Ms-admixed) was carried out on days 1 and 21 posttumor challenge. One group of mice received the anti-CD25 antibody injection (100 *μ*g), intraperitoneally on day 1 posttumor challenge. Mean tumor size ± SD (*n* = 5/group) over time is indicated for all groups (a). Animals that received the Ms-admixed vaccine and anti-CD25 antibody showed significantly slower tumor progression over time based on one-way ANOVA Bonferronis Post-test compared to the naïve group (*P* < .01). Tumor survival (b) is based on animals reaching a maximum tumor size of 300 mm^2^. Survival for the Ms-Admixed plus anti-CD25 antibody group was also significantly longer (*P* < .01) relative to the naïve group (*n* = 5 mice/group) based on Log rank test. The admixed group contained 2.2 mg of lipid and 60 *μ*g of peptide (30 *μ*g of each peptide).

Induction of prophylactic protection against cancer vaccines is often relatively easy to achieve, whereas breaking tolerance against an established tumor in a therapeutic setting can be challenging. Indeed, the admixed formulation of TRP- and Gp100-archaeosomes afforded only marginal protection (median survival of 24 days relative to median survival of 20 days for nonvaccinated group) when administered therapeutically following tumor challenge (Figure 7). However, when combined with prior depletion of T-regulatory cells using anti-CD25 antibody a significant decrease (*P* < .01) in tumor size (Figure 7(a)) and increased median survival (*P* < .01) were observed (Figure 7(b)). Thus, archaeosome vaccines appear to hold promise for tumor vaccination when combined with other vaccination strategies that target tumor evasion.

## 4. Discussion

Archaeosomes are effective self-adjuvanting delivery systems that target processing of antigenic cargo for MHC class I presentation leading to potent long-term CD8^+^ T cell responses. In previous studies using model antigens we demonstrated that following immunization of mice with Ovalbumin (OVA)-Archaeosomes, *∼*3.5% of all CD8^+^ T cells in the spleen were OVA-specific by day 7, and boosting on day 21 resulted in expansion to *∼*20% on day 28 [14]. Furthermore, a prolonged memory response ensued that was complemented with a strong functional cytolytic ability of CD8^+^ T cells and protection against OVA-expressing tumors in prophylactic and therapeutic settings [15]. Thus, we pursued the ability of archaeosomes to evoke adaptive immune responses to entrapped tumor-associated antigens. The generation of high frequencies of functional antigen-specific CD8^+^ T cells against two tumor-associated antigens, as assessed by IFN-*γ* secretion, enumeration of tetramer-binding antigen-specific CD8^+^ T cells, and *in vitro* and *in vivo* cytotoxic assays prove archaeosomes as a highly effective adjuvant for breaking tolerance to tumor self-antigens. 

Microparticulate carrier systems such as archaeosomes preferentially accumulate in APCs and thus can effectively target antigens for presentation onto MHC class I. However, secondary costimulatory signals are required for breaking tolerance to self-antigens which are often lacking in the context of tumors due to lack of danger-associated molecular patterns. Advantageously, archaeosomes exhibit the unique ability to induce dendritic cell maturation and increase costimulation [22]. Furthermore, archaeosomes evoke CD8^+^ T cell response in an IL-12-independent manner [15]. Thus, archaeosome vesicles uniquely bring together many beneficial features for tumor antigen presentation: antigen targeting to APC and costimulation in the absence of overt inflammation leading to effective adaptive immunity in a safe manner. 

Priming and boosting of an immune response has been traditionally recognized as an efficient way of maintaining long-term immunity. Indeed most vaccines against infectious diseases are often given multiple times and since priming and boosting occurs with the same vaccine, they are considered homologous prime-boost regimens. Heterologous prime-boosting with unmatched vaccine delivery systems or adjuvants but the same antigen was first reported in the early 1990s to be an effective strategy for evoking a balanced humoral and cell-mediated immunity [23]. Early studies focused on live viral vectors expressing antigens followed by a protein or peptide boost. With the advent of DNA vaccines that were weakly immunogenic, once again priming with DNA vaccine followed by boosting with a protein and peptide antigen proved to be effective strategy [24]. Since then, prime-boost regimens have been evaluated for several new generation vaccines against HIV, malaria, and tuberculosis. Heterologous prime-boost approaches can improve vaccine immunity by influencing the immunogenicity of antigens, allowing dose sparing, diversifying the quality of immunity, and circumventing the negative effects of neutralizing antibodies against the priming vector [9, 10, 24–26]. A heterologous prime-boost protocol involving different viral vectors expressing the same melanoma-polypeptide induced 100 times greater frequencies of vaccine-induced CD8^+^ T cells [27]. We thus rationalized that if a heterologous prime-boost regimes using model antigen-archaeosome vaccines were beneficial, this approach may be beneficial for cancer antigen delivery using archaeosomes. Our results indicate that while priming and boosting (2 injections) afforded superior tumor protection relative to a single injection regimen, there was no benefit to boosting with an alternative delivery system. The inefficiency of BCG-OVA to boost a good immune response may be related to its slow growth and low antigen expression. In contrast, 2 injections of Ms-OVA archaeosomes afforded superior tumor immunity comparable in efficacy to boosting with LM-OVA. Archaeosomes comprised of polar lipids of *M. smithii* do not usually evoke antilipid responses and hence demonstrate an advantage to be repeatedly utilized in vaccination regimens. 

Use of tumor antigenic peptides provides a quick, simple, and inexpensive manner of targeting induction of antitumor responses. T cell immunity against multiple antigenic determinants may be evoked. Peptides can be easily formulated to be tested in various settings, including altered peptide ligands, lipidated peptides, combination with heterologous helper peptides, and comparison of various adjuvants. Over the past decade, CTL epitopes of several melanoma antigens have been identified, and epitopes of melanoma antigens Gp100 and TRP-2 are widely targeted in clinical trials [4, 28–32]. Thus, we chose the use of these peptides for proof-of-concept studies to demonstrate the ability of archaeosomes to overcome self-tolerance. The overall negative charge of the total polar lipids mixture from *Methanobrevibacter smithii* with its high phospholipids content together with the charge/solubility of the peptides being used is major factors to consider during vaccine formulation. Each peptide type was solubilized and made into an appropriate overall charge and which had to be compatible with the hydration of the dry lipid film required for archaeosome formation. GP100 and OVA peptides were all more or less hydrophilic in character, whereas TRP-2 was more hydrophobic and required the addition of some propanol. TRP-2 peptide is acidic whereas GP100 peptide is basic. To avoid the aggregation of archaeosomes, a basic pH environment was favoured, and the lipid-peptide ratio was kept around 10 : 1 w/w. In this study, results are showing equivalent immune response using the admixed approach. The formulation of multiple peptide-archaeosome by admixing is appealing, since it requires optimization for only a single peptide at a time and may facilitate consistency of formulation and reproducibility. 

Vaccination with two melanoma antigen-archaeosomes afforded superior tumor protection in comparison to single antigen-archaeosome vaccines indicating that targeting responses to multiple antigens is an efficient way of breaking tolerance. Moreover coentrapment and/or admixture of individual peptide encapsulated archaeosomes afforded tumor protection. As archaeosomes are efficiently phagocytosed by antigen presenting cells (APC), coentrapped antigens within the same vesicle may be targeted for release within the same APC. Once released in the intracellular milieu, peptides may compete for binding to the transporter associated with antigen processing (TAP), and differential binding of melanoma peptides to TAP has been reported [33, 34]. The differential CTL response to the two peptides following coentrapment may be reflective of their differential binding to TAP or just difference in the amount of each peptide that was coentrapped. Moreover, peptides may also compete for binding to the same MHC molecules, and epitopes with higher avidity for the MHC-I complex may outwit weakly binding peptides. In contrast, when peptides are individually entrapped and used in an admixed formulation, each antigen is targeted individually for presentation by the APC. Furthermore, optimized amounts of each antigen may be included in the vaccine. Our results demonstrate that admixed peptide-archaeosome formulations induced CTL responses against both epitopes. It has been previously shown that recognition of melanoma CTL epitopes with different affinities can be achieved following multiple peptide formulation [35]. Nevertheless, even with the admixed vaccine, responses to the TRP-2 peptide was stronger based on frequency of IFN-gamma secreting cells, tetramer^+^ CD8^+^ T cell number, and CTL response. Indeed, it has been suggested that with multiple peptide vaccinations, competition between CTLs can narrow the repertoire of the immune response evoked [27]. Despite such a possibility, multiple peptide immunization often provides a cumulatively stronger overall CD8^+^ CTL response [36]. Indeed, the dual-peptide archaeosome vaccine afforded longer-term tumor protection relative to the single-peptide archaeosome formulation. 

A number of particulate delivery systems have been evaluated for cancer vaccine delivery. Conventional liposomes carrying cytokines such as IL-2 and IFN-gamma were tested for their efficacy to deliver cancer antigens but showed limited promise in breaking tolerance [37, 38]. Cationic liposomes posed the ease for efficient association with diverse negatively charged antigens including DNA [39] but their high positive charge and large size lead to quick clearance from the blood and to tissue toxicity. More recently, lipid particle-based nanovesicles with neutral charge were utilized to deliver negatively charged CpG oligonucleotides in a safe and consistent manner [40]. A novel proteoliposomal vaccine prepared from the cell membrane proteins of lymphoma cells was also shown to be more efficient in inducing antitumor responses than conventional liposomes [41]. ISCOM vaccines that combine TLR9 agonists have been reported to break tolerance in an orthotopic model of pancreatic carcinoma [42]. 

In many cases combination immunotherapy includes novel vaccination regimens to overcome immunologic tolerance. A synthetic TRP2_180–188_ peptide vaccine was recently combined with several toll-like receptor agonists and an anti-CD40 antibody to induce potent CD8^+^ T cells with therapeutic tumor efficacy against established B16 melanoma [43]. Other strategies to counteract tumor evasion and promote long-term tumor recession have included blocking T regulatory cells and programmed death-ligand interactions, or co-use of adoptive T cell transfer [44, 45]. We also observed that while both Gp100- and TRP-2-archaeosome vaccines evoked antigen-specific CD8^+^ T cell response indicating their ability to break tolerance, tumor protection against established tumors required depletion of T regulatory cells. Indeed, it is now well established that T regulatory cells can hinder the protective efficacy of tumor-specific CD8^+^ T cells. An anti-CD25 monoclonal antibody, daclizumab, has been effectively trialed for the depletion of T-regulatory cells and improved efficacy of a coadministered peptide-vaccine against human breast cancer [46]. Our studies reiterate that induction of functional CD8^+^ T cells alone is an insufficient marker for predicting tumor protection and immunotherapy may require additional strategies to counteract tumor evasion. Thus, archaeosomes should be further evaluated as a promising adjuvant delivery system for immunotherapeutic vaccination regimens.
